# Loss of Y-Chromosome during Male Breast Carcinogenesis

**DOI:** 10.3390/cancers12030631

**Published:** 2020-03-09

**Authors:** Marie Colombe Agahozo, Mieke A. M. Timmermans, Hein F. B. M. Sleddens, Renée Foekens, Anita M. A. C. Trapman-Jansen, Carolien P. Schröder, Elise van Leeuwen-Stok, John W. M. Martens, Winand N. M. Dinjens, Carolien H. M. van Deurzen

**Affiliations:** 1 Department of Pathology, Erasmus MC Cancer Institute, 3015 GD Rotterdam, The Netherlands; m.agahozo@erasmusmc.nl (M.C.A.); h.sleddens@erasmusmc.nl (H.F.B.M.S.); w.dinjens@erasmusmc.nl (W.N.M.D.); 2 Department of Medical Oncology, Erasmus MC Cancer Institute, 3015GD Rotterdam, The Netherlands; a.timmermans@erasmusmc.nl (M.A.M.T.); r.foekens@erasmusmc.nl (R.F.); a.trapman@erasmusmc.nl (A.M.A.C.T.-J.); j.martens@erasmusmc.nl (J.W.M.M.); 3 Department of Medical Oncology, University Medical Center Groningen, 9700AB Groningen, The Netherlands; 4 Dutch Breast Cancer Research Group, BOOG Study Center, 1006 AE Amsterdam, The Netherlands; e.vanleeuwen@boogstudycenter.nl

**Keywords:** male breast cancer, loss of Y-chromosome, ductal carcinoma in situ, invasive breast cancer, progression

## Abstract

Loss of Y-chromosome (LOY) is associated with increased cancer mortality in males. The prevalence of LOY in male breast cancer (BC) is unknown. The aim of this study is to assess the presence and prognostic effect of LOY during male BC progression. We included male BC patients diagnosed between 1989 and 2009 (n = 796). A tissue microarray (TMA) was constructed to perform immunohistochemistry and fluorescent in situ hybridization (FISH), using an X and Y probe. We also performed this FISH on a selected number of patients using whole tissue slides to study LOY during progression from ductal carcinoma in situ (DCIS) to invasive BC. In total, LOY was present in 12.7% (n = 92) of cases, whereby LOY was associated with ER and PR negative tumors (*p* = 0.017 and *p* = 0.01). LOY was not associated with the outcome. Using whole slides including invasive BC and adjacent DCIS (n = 22), we detected a concordant LOY status between both components in 17 patients. In conclusion, LOY is an early event in male breast carcinogenesis, which generally starts at the DCIS stage and is associated with ER and PR negative tumors.

## 1. Introduction

Male breast cancer (BC) is a relatively rare disease that accounts for less than 1% of all BCs [1,2]. It is generally diagnosed at a later stage compared to female BC, which worsens the outcome [2,3]. Male BC is generally estrogen-receptor (ER) positive, progesterone receptor (PgR) positive, androgen receptor (AR) positive, and human epidermal growth factor receptor 2 (HER2) negative [1,3,4,5]. Historically, male BC literature was restricted to small single-center studies, thus data regarding male BC biology are relatively limited.

Loss of Y-chromosome (LOY) has previously been described in several solid tumors, including esophageal carcinoma, pancreatic cancer, urothelial bladder cancer, colorectal cancer, and prostate cancer [6,7,8,9,10,11]. Early case reports described the presence of LOY in male BC tissue [10,11,12]. A larger, more recent study reported LOY in 5 out of 31 patients with male BC. They suggested that LOY could lead to the loss of a candidate tumor suppressor gene (TMSB4Y), resulting in increased cell proliferation [13]. Additionally, they reported LOY in the corresponding ductal carcinoma in situ (DCIS) component in one male BC case, suggesting that LOY could be an early event in male breast carcinogenesis [13]. In the peripheral blood of males, LOY has been reported to be associated with an increased risk of all-cause mortality and non-hematological cancer mortality [14]. Although this study does not mention male BC, they conclude that LOY could become a predictive biomarker for male carcinogenesis. Nonetheless, the presence and role of LOY during breast carcinogenesis remain poorly understood.

The aim of this study was, therefore, to assess the presence and potential prognostic effect of LOY in male BC using a large cohort of patients. Secondary, we studied the presence of LOY during the progression from DCIS to invasive BC by performing fluorescent in situ hybridization (FISH) on paired DCIS and invasive BC.

## 2. Results

### 2.1. General Patients and Tumor Characteristics

A total number of 796 patients were included. Table 1 includes general clinicopathological features of this cohort. The median age at diagnosis was 67 years (range 25 to 98 years). Within this cohort, 46.0% of cases had a breast cancer precursor lesion adjacent to the invasive component. The majority of these precursor lesions (98.4%) were classified as DCIS. Median follow up time was 89 months (range 0 to 323 months), in which 62.1% of the patients died. Recorded breast cancer specific survival was available for only 96 patients, of which 49 patients died due to breast cancer progression.

### 2.2. LOY in Male BC and Outcome

The presence of LOY was detected in 12.7% (92 out of 722) of patients. These patients had a median age of 64 years (range 34-98 years). There was a significant association between LOY and ER and PR status (*p* = 0.017 and *p* = 0.01, respectively), whereby LOY was associated with ER and PR negative tumors. There was no significant association between LOY and other tumor characteristics, although there was a trend for an association with tumor grade (*p* = 0.056) (Table 2). In addition, there was no association between LOY and overall survival, recurrence-free survival or breast cancer-specific survival (Hazard Ratio: 1.23 (95% Confidence Interval (CI) 0.86–1.48), 1.12 (95%CI 0.86–01.46) and 0.47 (95%CI 0.17–1.33) respectively). 

### 2.3. LOY during Progression from DCIS to Invasive BC

Out of the 92 patients with LOY based on TMA, 40 had an adjacent DCIS component in the whole tissue slide. In 22 patients, LOY was analyzed on whole tissue slides, including DCIS and adjacent invasive disease (remaining 18 cases were excluded due to limited tissue availability). Table 3A depicts the results of the whole slide analysis of these 22 patients. All invasive BC cases (n = 22) with LOY based on TMA also had LOY in the whole tissue slide. We did not find LOY in non-tumor breast tissue.

**Figure 1:**
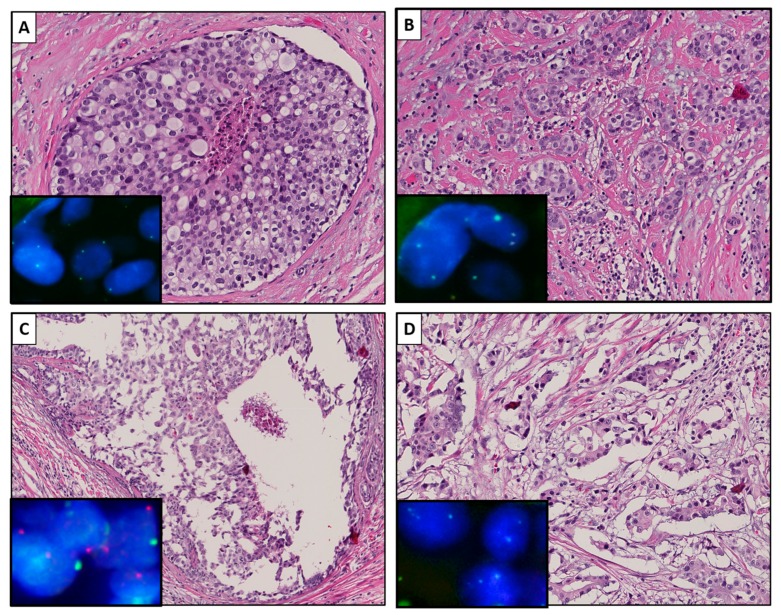
Representative images of a male breast cancer (BC) case with a concordant loss of Y-chromosome (LOY) status between ductal carcinoma in situ (DCIS) (**A**) and invasive BC (**B**) and a case with a discordant LOY status between DCIS (**C**) and invasive BC (**D**). Hematoxylin and eosin staining, at a 15× magnification and insets of the corresponding XY fluorescent in situ hybridization (FISH) images at a 63× magnification. The cell nuclei are depicted in blue (dapi), the X-chromosome is depicted in green (Fluorescein-5-isothiocynate;FITC), and the Y-chromosome is depicted in red (Texas red).

A concordant LOY status between IBC and adjacent DCIS was found in 17 out of 22 patients. In these 17 patients, LOY was detected in both DCIS and invasive BC. A discordant LOY status was detected in four patients. LOY was detected in these four invasive BCs, whereby the corresponding DCIS component did not have LOY. The LOY status of the DCIS component of one case remained undetermined. Figure 1 illustrates a case with a concordant (A and B) and a discordant (C and D) status between the DCIS and the invasive component, respectively.

From patients without LOY in the invasive component based on TMA, we selected 20 cases with adjacent DCIS in the whole tissue slide. None of these cases had LOY, neither in the invasive component nor the DCIS component. Table 3B depicts the results of the whole slide analysis of the 20 patients without LOY.

## 3. Discussion

The aim of our study was to assess the presence and prognostic effect of LOY during male BC progression. Our study is the first to describe LOY in a large cohort of male BC patients. Previous studies of LOY in male BC were restricted to blood samples, analyses of the invasive component only, or included small numbers [10,11,12,13]. In the current study, LOY was detected in 12.7% of male BC cases, whereby tumors with LOY were more likely to be ER and/or PR negative. This association suggests that patients with LOY might have more aggressive tumors since ER-negative breast tumors are generally associated with shorter overall survival [3,4,15]. Additionally, the presence of LOY in peripheral blood was also recently associated with all-cause mortality and non-hematological cancer mortality [14,16]. However, in our series, there was no association between LOY and survival. A possible explanation for the lack of association between LOY and outcome in our series are missing breast cancer-specific outcome data for the majority of patients in this cohort. Furthermore, our data were restricted to the presence of LOY in breast cancer tissues, whereas data regarding LOY in peripheral blood cells was missing.

In our series of invasive male BC samples with LOY, about 43% were associated with an adjacent DCIS component. We did not detect any LOY in non-tumor breast epithelia by XY analysis on whole tissue slides. This is consistent with the results of Wong and colleagues, who also demonstrated that LOY was restricted to malignant lesions [13]. We detected LOY in the DCIS component in the majority (17 out of 22) of cases with LOY in the invasive component. This suggests that LOY in male BC tissue is a process that is mostly already present in the DCIS component, resulting in LOY in the invasive component. Furthermore, this further supports that DCIS is a precursor lesion of invasive male BC, which is in line with other studies that demonstrated similar molecular aberrations in DCIS and paired invasive male BC [17,18]. This early role for LOY in male carcinogenesis was also previously suggested by Wong and colleagues [13]. Using a functional assay, they showed that clonal LOY contributes to breast carcinogenesis through the deletion of a Y-chromosome expressed tumor suppressor gene. Together with our data, showing an early LOY, this suggests that LOY might contribute to male breast carcinogenesis through dysregulation of the cell proliferation and differentiation mechanism.

The strength of our study is the size of our cohort. This study is by far the largest study reporting on LOY in male BC, including the evolution of LOY during the progression of DCIS to invasive BC. However, our study also had several limitations. First, outcome data are incomplete, as mentioned above, which limits the analyses of the clinical impact of LOY. Second, we included BC samples between 1989 and 2009, thus a substantial proportion of samples were relatively old. This hampered the FISH analysis, resulting in the exclusion of 9.3% of cases due to undetermined XY status. Future studies could also use in vitro models to confirm our findings. However, male BC is rare, making the use of in vitro models, specifically for DCIS, challenging. Additionally, a comprehensive and integrated genomic analysis could be performed to shed more light on the molecular mechanisms that affect or might be affected by LOY in male BC.

## 4. Materials and Methods

### 4.1. Patients

This work was approved by the Medical Ethics Committee of the Erasmus MC (approval number MEC 02.953). According to national guidelines, no informed consent was needed for this study. This study included all Dutch male BC cases diagnosed with invasive BC between 1989 and 2009. Central pathology review was performed based on whole tissue slides, including histologic subtype, grade (according to Bloom and Richardson), the presence and type of BC precursor lesions, and density of tumor-infiltrating lymphocytes (TILs), as described previously [5,19,20]. Other clinicopathological data were collected by the Netherlands Comprehensive Cancer Organization (IKNL), including age at diagnosis, tumor size, nodal status, and outcome.

Overall survival was defined as the time between initial diagnosis and death due to any cause. Relapse free survival was defined as the time between initial diagnosis and ipsilateral recurrence, metastasis, or death due to any cause. Breast cancer specific survival was defined as the time between diagnosis and breast cancer specific death.

### 4.2. Immunohistochemistry on Tissue Micro-Array

A tissue microarray (TMA) of all invasive male BC cases was constructed and used to assess ER, PR, AR, and HER2 status. An overview of these antibodies is depicted in Table 4. Briefly, 4 µm-thick formalin-fixed paraffin-embedded (FFPE) TMA slides were dewaxed, and heat-induced antigen retrieval was performed at antibody specific pH, varying from 6.0 to 9.0. The tissue samples were then incubated with the primary antibody, followed by a hematoxylin counterstain, whereby DAB was used as a chromogen. ER and PR status was classified as positive when the percentage of positive tumor cells was at least 10%, according to Dutch guidelines [21]. The cut-off for AR positivity was also set at 10% [3]. HER2 status was scored according to international guidelines [22]. For this study, coded leftover patient material was used in accordance with the Code of Conduct of the Federation of Medical Scientific Societies in The Netherlands [23].

### 4.3. Fluorescent In Situ Hybridization on Invasive BC Using Tissue Micro-Array

We performed an XY specific FISH on a TMA of invasive male BC to determine LOY using the Satellite Enumeration (SE) X (DXZ1)/ Y (DYZ3) FISH probe (catalog number: PI-KBI-20030 D1.1, Kreatech^TM^ FISH probes, Leica Biosystems, Wetzlar, Germany). This dual-color probe contained a green-labeled (DXZ1) probe for the X chromosome (at Xp11.1-q11.1 with PlatinumBright ^TM^ 495) and a red-labeled (DYZ3) probe for the Y chromosome, (at Yp11.1-q11.1 with PlatinumBright ^TM^ 550). First, 4 µm-thick FFPE TMA slides were dewaxed and dehydrated, cooked for 13 min in citrate buffer, and then treated with pepsin for 20 min. Hereafter, hybridization followed first at 75 °C for 10 min and then at 37 °C overnight in a Hybridizer (Dako Agilent, Stanta Clara, CA, United States). Non-specific binding was removed by a stringent wash buffer at 73 °C. The slides were rinsed in a 2× SCC, dehydrated, air-dried, and sealed with a cover glass. They were stored at 4 °C until further use.

**Figure 2:**
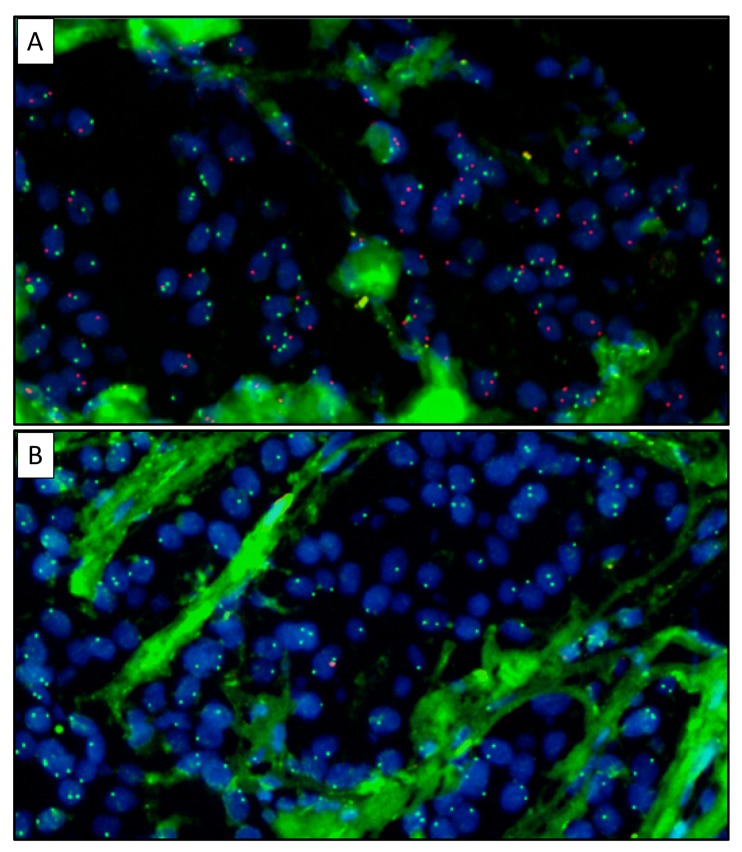
Representative images of male BC cases with XY (**A**) and LOY (**B**), both at a 40× magnification. The cell nuclei are depicted in blue (dapi), the X-chromosome is depicted in green (FITC), and the Y-chromosome is depicted in red (Texas red).

For visualization and analysis, the stained slides were scanned by the Vectra 3 automated quantitative pathology imaging system (Akoya biosciences, Malborough, MA, USA). Selected cores were manually scored using Inform (Akoya biosciences, Malborough, MA, USA), whereby LOY was defined as the absence of Y-chromosome in at least 75% of tumor cells. Figure 2 depicts representative images scored as XY (A) or LOY (B).

### 4.4. Fluorescent In Situ Hybridization on Invasive BC and Adjacent DCIS Using Whole Tissue Slides

**Figure 3:**
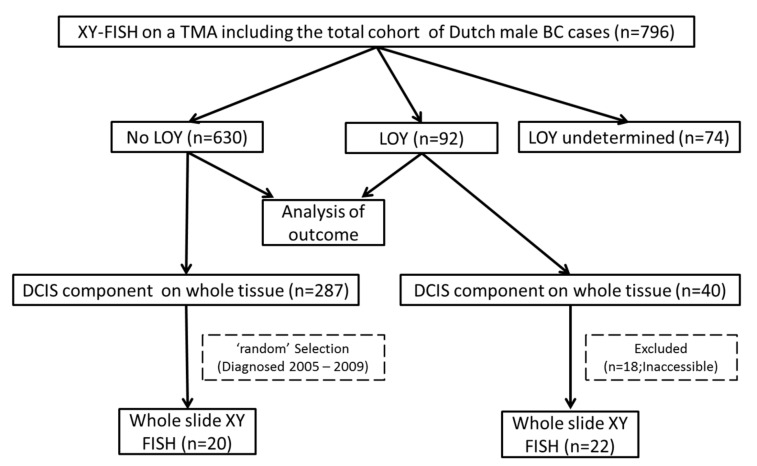
Study flowchart. TMA: tissue microarray.

In total, 22 cases with LOY and 20 cases without LOY in the invasive component on TMA were selected for further analyses to study the pattern of LOY during progression from DCIS to invasive BC. For this purpose, we included patients with a DCIS component in the whole tissue section. The XY FISH was performed on whole tissue sections to assess the presence of LOY in paired DCIS and invasive BC cells. Figure 3 depicts the workflow of our study.

### 4.5. Statistical Analysis

The Chi-square test was used to analyze associations between LOY and clinicopathological features. The Mann-Whitney U test was used to compare continuous variables between patients with LOY and those without LOY. A Cox proportional hazards regression was used to examine the overall survival, recurrence-free survival, and breast cancer-specific survival. *p*-values < 0.05 were considered significant.

## 5. Conclusions

In conclusion, we demonstrated that LOY is present in a substantial proportion (12.7%) of male BC cases and that it was associated with ER and PR negative tumors. With regard to progression, there was a concordant LOY status between the DCIS component and the paired invasive component in the majority of cases. We, therefore, suggest that LOY is an early event, which starts in the DCIS stage and mostly results in LOY at the invasive stage.

## Figures and Tables

**Table 1:** Clinic pathological baseline characteristics (n = 796).

Patients and Tumor Characteristics	n	Range/%
Age at diagnosis (in years)		
Median-range	67	25–98
Tumor size (in mm) (missing; n = 413)		
Median-range	20	1–110
Grade (%) (missing; n = 30)		
Low	190	24.8
Intermediate	401	52.3
High	175	22.8
Precursor lesion (missing; n = 27)		
None	403	52.4
DCIS	360	46.8
Other	6	0.8
TIL density (missing; n = 32)		
Minimal-Mild	652	81.9
Moderate-Severe	112	14.1
ER status (missing; n = 54)		
ER+	694	93.5
ER−	48	6.5
PR status (missing; n = 42)		
PR+	548	72.7
PR−	206	27.3
AR status (missing; n = 45)		
AR+	533	71.0
AR−	218	29.0
HER2 status (missing; n = 24)		
HER2+	34	4.4
HER2−	738	95.6
IHC subtype (Undermined; n = 77)		
ER+PR+/-HER2−	649	90.3
ER+PR+HER2+	32	4.5
ER-PR-HER2+	1	0.1
ER-PR-HER2−	37	5.1
Loss of Y (Undetermined; n = 74)		
XY	630	87.3
X_	92	12.7
Distant metastasis (missing; n = 494)		
Yes	66	21.9
No	236	78.1
Survival (missing; n = 6)		
Alive	296	37.5
Dead	494	62.5

**Table 2:** Association of loss of Y-chromosome (LOY) with male breast cancer (BC) clinicopathological characteristics (n = 722).

Patient and Tumor Characteristics	XY Status		*p*-Value
	XY (Range/%)	X_ (Range/%)	
Age at diagnosis			0.311
Median (years)	67.0 (25–95)	64.5 (34–98)	
Tumor size (missing; n = 362)			0.093
Median (mm)	20.0 (1–110)	21.0 (0–90)	
Grade (missing; n = 26)			0.056
Low	161 (26.5)	13 (14.7)	
Intermediate	306 (50.3)	53 (60.2)	
High	141 (23.2)	22 (25.0)	
Precursor lesion (missing; n = 24)			0.605
None	317 (52.0)	48 (54.5)	
DCIS	287 (47.0)	40 (45.5)	
Other	6 (1.0)	0 (0.0)	
TIL density (missing; n = 28)			0.268
Minimal-Mild	515 (85.0)	13 (54.2)	
Moderate-Severe	91 (15.0)	11 (45.8)	
ER status (missing; n = 41)			0.017
ER+	561 (94.4)	76 (87.4)	
ER−	33 (5.6)	11 (12.6)	
PR status (missing; n = 34)			0.01
PR+	448 (74.4)	53 (61.6)	
PR−	154 (24.6)	33 (38.4)	
AR status (missing; n = 32)			0.327
AR+	428 (67.9)	67 (72.8)	
AR−	174 (27.6)	21 (22.8)	
HER2 status (missing; n = 17)			0.542
HER2+	29 (4.7)	3 (3.3)	
HER2−	585 (95.3)	88 (96.7)	
IHC subtype (undetermined; n = 60)			0.242
ER+/-PR+/-HER2−	525 (90.7)	72 (86.7)	
ER+PR+/-HER2+	27 (4.6)	3 (3.6)	
ER-PR-HER2+	1 (0.2)	0 (0.0)	
ER-PR-HER2−	26 (4.5)	8 (9.6)	
Distant metastasis (missing; n = 451)			0.157
Yes	54 (22.6)	3 (10.8)	
No	189 (79.4)	25 (89.2)	
Survival (missing; n = 5)			0.469
Alive	240 (38.4)	30 (33.0)	
Dead	386 (61.6)	61 (67.0)	

**Table 3:** Whole slide LOY analysis of patients with invasive BC and adjacent ductal carcinoma in situ (DCIS).

Patient	DCIS	Invasive BC
**A: Patients with LOY in the Invasive Component (n = 22)**		
1	X_	X_
2	X_	X_
3	X_	X_
4	X_	X_
5	XY	X_
6	X_	X_
7	X_	X_
8	X_	X_
9	X_	X_
10	XY	X_
11	X_	X_
12	XY	X_
13	X_	X_
14	Undetermined	X_
15	X_	X_
16	X_	X_
17	X_	X_
18	X_	X_
19	X_	X_
20	X_	X_
21	X_	X_
22	XY	X_
**B: Patients without LOY in the invasive component (n = 20)**		
1	XY	XY
2	XY	XY
3	XY	XY
4	XY	XY
5	XY	XY
6	XY	XY
7	XY	XY
8	XY	XY
9	XY	XY
10	XY	XY
11	XY	XY
12	XY	XY
13	XY	XY
14	XY	XY
15	XY	XY
16	XY	XY
17	XY	XY
18	XY	XY
19	XY	XY
20	XY	XY

**Table 4:** Antibody characteristics and used protocol for immunohistochemistry.

Antibody	Type	Company	Clone	Lot Number	Dilution	Antigen Retrieval pH	Incubation Time
ER	Anti-mouse	Dako	1D5	M7047	1:40	9	60 min
PR	Anti-mouse	Dako	PgR 636	M3569	1:50	9	60 min
AR	Anti-mouse	ErasmusMC	F39.4	Trapman	1:50	9	Overnight
HER2neu	Anti-Rabbit	Dako	Herceptest	K5204	ready to use	ready to use	60 min
